# Multimodal approaches for the improvement of the cellular folding of a recombinant iron regulatory protein in *E. coli*

**DOI:** 10.1186/s12934-022-01749-w

**Published:** 2022-02-05

**Authors:** Gayathri Ravitchandirane, Sheetal Bandhu, Tapan K. Chaudhuri

**Affiliations:** 1 Kusuma School of Biological Sciences, Indian Institute of Technology Delhi, Hauz Khas, New Delhi, 110016 India; 2 Dr. Reddy’s Laboratories Ltd., Hyderabad, India

**Keywords:** Cellular protein folding, Protein aggregation, Yeast mitochondrial aconitase, Molecular chaperone, Cellular stress, GroEL/ES, Inclusion bodies

## Abstract

**Background:**

During the recombinant protein expression, most heterologous proteins expressed in *E. coli* cell factories are generated as insoluble and inactive aggregates, which prohibit *E. coli* from being employed as an expression host despite its numerous advantages and ease of use. The yeast mitochondrial aconitase protein, which has a tendency to aggregate when expressed in *E. coli* cells in the absence of heterologous chaperones GroEL/ES was utilised as a model to investigate how the modulation of physiological stimuli in the host cell can increase protein solubility. The presence of folding modulators such as exogenous molecular chaperones or osmolytes, as well as process variables such as incubation temperature, inducer concentrations, growth media are all important for cellular folding and are investigated in this study. This study also investigated how the cell's stress response system activates and protects the proteins from aggregation.

**Results:**

The cells exposed to osmolytes plus a pre-induction heat shock showed a substantial increase in recombinant aconitase activity when combined with modulation of process conditions. The concomitant GroEL/ES expression further assists the folding of these soluble aggregates and increases the functional protein molecules in the cytoplasm of the recombinant *E. coli* cells.

**Conclusions:**

The recombinant *E. coli* cells enduring physiological stress provide a cytosolic environment for the enhancement in the solubility and activity of the recombinant proteins. GroEL/ES-expressing cells not only aided in the folding of recombinant proteins, but also had an effect on the physiology of the expression host. The improvement in the specific growth rate and aconitase production during chaperone GroEL/ES co-expression is attributed to the reduction in overall cellular stress caused by the expression host's aggregation-prone recombinant protein expression.

**Graphical Abstract:**

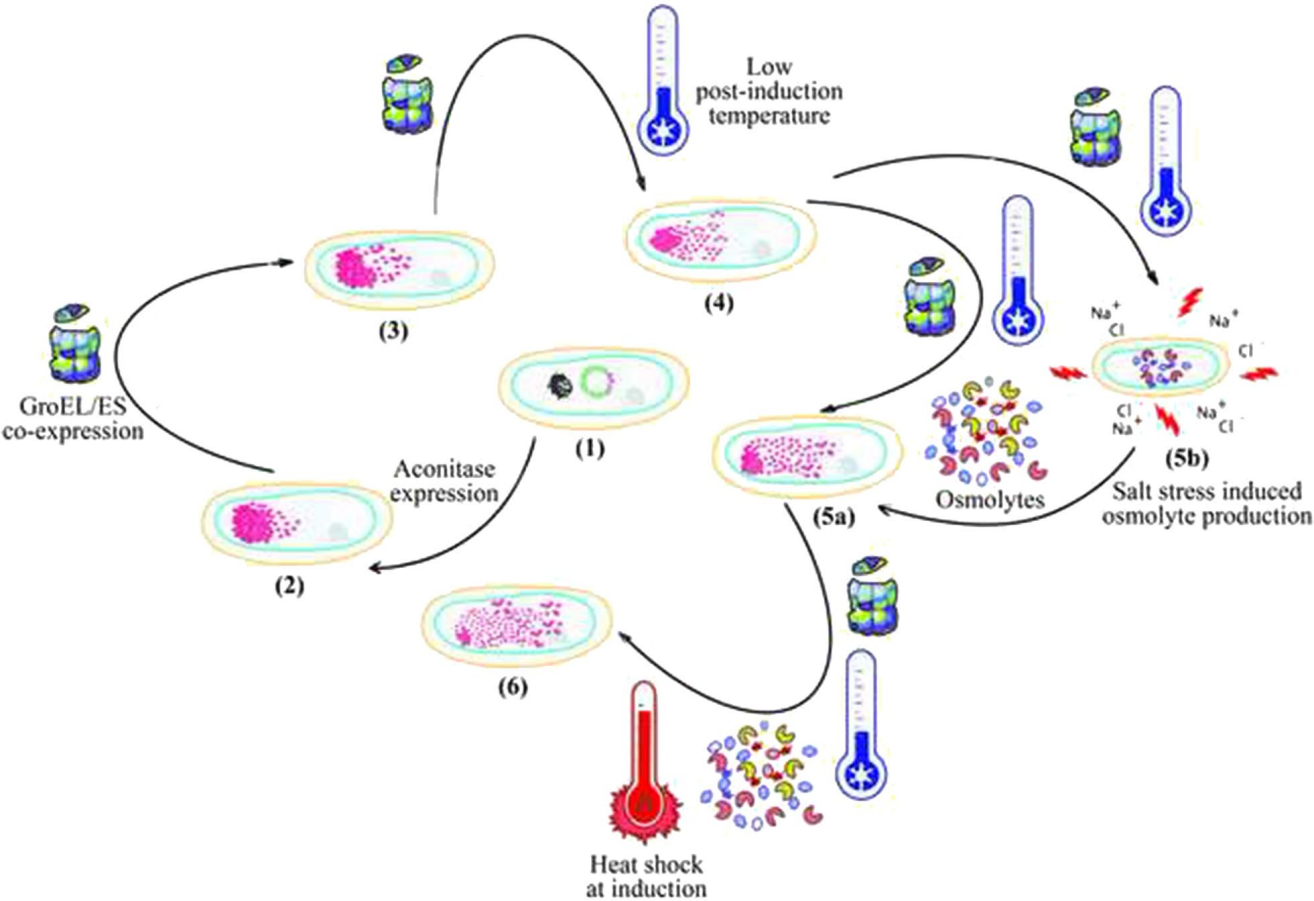

**Supplementary Information:**

The online version contains supplementary material available at 10.1186/s12934-022-01749-w.

## Background

Proteins are the molecular machineries that possess biological functionality and often find indispensable roles in the fields of cosmetics, biofuels and pharmaceuticals. Owing to the advanced developments in the recombinant DNA (rDNA) technology, gene of any length and origin can now be expressed in any of the expression systems like animals, plants and microorganisms. The combination of rDNA technology and the scale-up processes provide a platform for large-scale production of recombinant proteins to meet the growing demands. Among the different expression hosts, *E. coli* is the most extensively employed prokaryotic expression host, accounting for 30% of all recombinant biopharmaceutical goods approved by the FDA and EMEA [1]. Its properties like faster multiplication rates, growth on cheap substrates, availability of tools for genetic manipulation, etc. favour efficient expression of recombinant proteins at industrial scales [2]. However, high protein expression is not enough, in order to function, the nascent polypeptides have to attain a precise complex structural confirmation by a process called as protein folding. The nascent polypeptides that fail to attain the native three-dimensional structural confirmation are termed as misfolded proteins. Misfolding can result due to mutations in the gene, translational errors, absence of post-translational machinery and other cellular stresses resulting in the loss of protein’s native structure [3]. The misfolded proteins lose their functionality and expose the intricate core hydrophobic stretches of residues that are usually buried within the native structure. These exposed hydrophobic protein moieties associate with one another through weak forces of attraction and form aggregates which act as seeding steps for the large-scale aggregation process leading to the formation of insoluble aggregates known as inclusion bodies [3, 4]. Without defined techniques for protein refolding, recovery is frequently done on a trial-and-error basis, making protein production a time-consuming and costly process. The propensity of *E. coli* to generate protein aggregates is an obstacle to its utilisation as a host despite its several advantages.

In recombinant cells, the high translational rates and cellular crowding due to protein over-expression together with the reducing cellular environment leads to protein misfolding. The genetic manipulations also influence the physiological characteristics of the cellular functions challenging the recombinant cells with increased metabolic burden and cellular stress that also promotes protein aggregation and significantly impedes the growth [5]. *E. coli* overcomes the aforesaid hurdles by activating its innate defensive systems in response to diverse physiological stresses. The common intrinsic recovery mechanism involves expression of specific sigma factors. These are tiny proteins that bind to RNA polymerase and direct them to bind specific promoters and induce expression of heat shock proteins like DnaK-DnaJ-GrpE and GroEL/ES [6, 7]. These proteins function by facilitating the refolding of aggregated proteins. The heat shock proteins, which are also called as the molecular chaperones, are the giant molecular assemblies which interact with individual misfolded proteins to assist in its folding for the attainment of the native structure [8]. GroEL/ES is one of the prokaryotic chaperone machinery comprising of the chaperonins GroEL and its co-chaperone GroES, which assists the folding of the misfolded proteins by providing a cage-like enclosure away from the crowded cellular environment [9]. However, the physiological concentrations of molecular chaperones in host organisms are insufficient to accommodate the enormous amount of misfolded recombinant proteins produced. Hence, the co-expression of higher amounts of molecular chaperones from exogenous plasmids regulated by a strong promoter must help in folding the misfolded polypeptides and improve the solubility of the proteins [10].

Modulation of environmental conditions such as temperature, inducer concentration and the medium are known to affect the protein solubility [11]. The cells grown under physiological stress conditions including osmotic and heat-shock stresses adapt to the environment to maintain normal cellular function. In order to overcome physiological stresses, the bacterial system adapts itself to synthesize bio-molecules and endogenous heat-shock proteins that can evade the harsh effects and help in normal functioning of the cells. It is now known that certain stresses elicit similar global stress responses, and that adaptation to one form of stress can lead to resistance to another [12]. In this work, we have exploited these features of the bacterial cells grown under stress conditions to improve the solubility and activity of the aggregation-prone recombinant protein. It has also been a focus of research to investigate how cells endure osmotic stress and preserve cell homeostasis by creating osmolytes, which help proteins fold properly by stabilising their structure. We used a bench-scale screening platform to optimise the productivity, solubility, and activity of recombinant yeast mitochondrial aconitase, through application of GroEL/ES assisted folding process in *E. coli*. The mature yeast mitochondrial aconitase is an 82 kDa monomeric metalloenzyme with Fe_4_S_4_ cluster as the prosthetic group. It catalyzes one of the tricarboxylic acid cycle reactions, the isomerization of citrate to iso-citrate in the presence of Fe_4_S_4_ acting as the prosthetic group. In this study, aconitase was taken as the model protein as it is an obligate substrate for GroEL/ES chaperone and tends to aggregate when expressed in *E. coli* in absence of GroEL/ES [13]. Although, the role of GroEL/ES in improving the solubility of recombinant protein has been reported elsewhere, in the present work we have not only shown the co-expressed chaperone assisted improvement of recombinant protein folding, but also demonstrated how the cellular folding of recombinant aconitase has been improved through combinatorial effect of chaperone co-expression, use of osmolytes, and induced cellular stress response.

## Results

### Cloning of mature yeast mitochondrial aconitase and co-expression with GroEL/ES chaperones

**Fig. 1:**
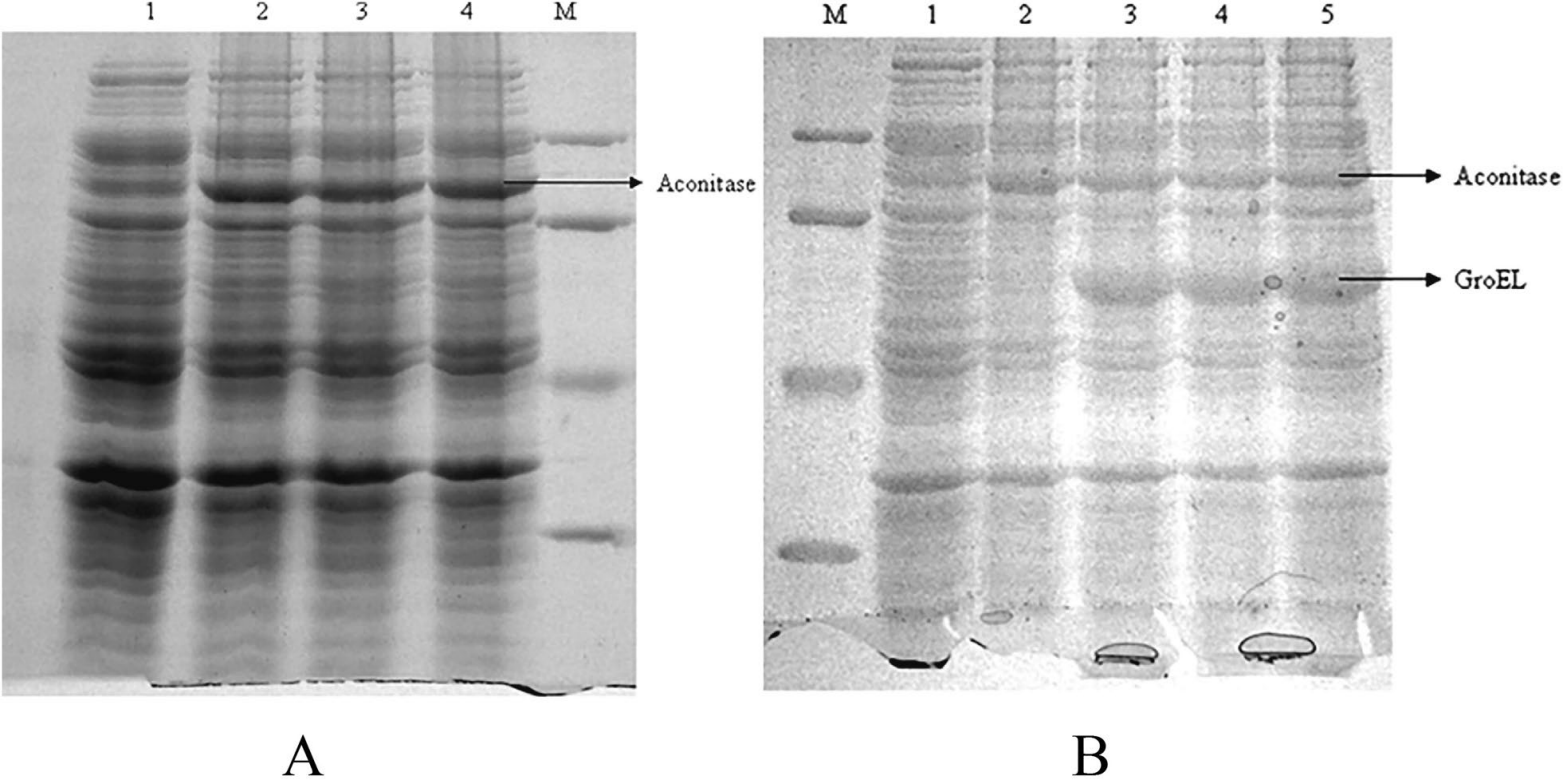
**A** BL21(DE3) cells transformed with pETAco plasmid expressing aconitase; Lane M: Protein marker; Lane 1: Uninduced cells; Lanes 2 to 4: Cells induced to express aconitase. **B** BL21(DE3) cells co-transformed with plasmids pETAco and pGro7 expressing proteins aconitase and GroEL/ES respectively; Lane M: Protein marker; Lane 1: Uninduced cells; Lane 2: Cells induced to express only aconitase; Lanes 3 to 4: Cells induced to express aconitase and Gro EL/ES

A 2.4 kb mature yeast mitochondrial aconitase (*aco1)* gene was amplified from the template plasmid pQE60Aco by polymerase chain reaction and cloned downstream of the T7*lac* promoter in pET29 vector and the resulting plasmid was designated as pETAco. The insertion of the mature aconitase gene was confirmed by restriction digestion and gene sequencing (Additional file 1: Fig S1). Both results confirmed the cloning of the mature aconitase gene sequence in the pET29 vector. The total aconitase protein expression was checked on SDS-PAGE gel of the BL21(DE3) cells transformed with the plasmid pETAco, grown in LB media until mid-log phase and induced with 0.5 mM IPTG (Fig. 1A). The cells expressing aconitase were re-transformed with pGro7 for expression of GroEL/ES. The co-expression of both the yeast mitochondrial aconitase and GroEL/ES was verified on a 12% SDS-PAGE gel of total cell lysate from transformed BL21(DE3) cells induced with 0.5 mM of sterile IPTG (for expression of aconitase) and 0.5 g/L of l-arabinose (for expression of chaperones GroEL/ES) (Fig. 1B).

### Effect of temperatures during chaperone-assisted folding in *E. coli* cells

**Fig. 2:**
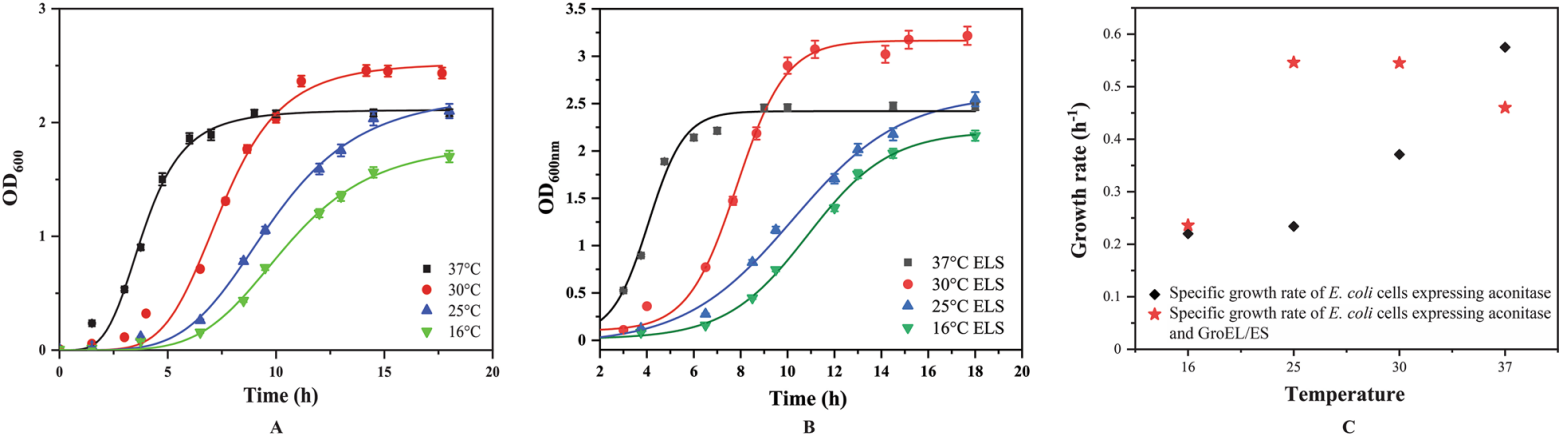
The influence of different incubation temperatures on the biomass profiles of induced BL21(DE3) cells transformed with plasmids. **A** Growth profile of *E. coli* cells expressing aconitase at different temperatures. **B** Growth profile of *E. coli* cells expressing aconitase and GroEL/ES. **C** Specific growth rate of *E. coli* cells expressing aconitase at different temperatures in presence and absence of GroEL/ES

Micro-organisms require an optimal temperature for growth below which the growth rate tends to decrease. When the BL21(DE3) cells producing recombinant aconitase were grown at 37 °C, 30 °C, 25 °C and 16 °C, a steady decline in the specific growth rate was observed with decrease in temperatures demonstrating longer lag phase (Fig. 2A). The biomass concentration of the cells expressing only aconitase at all temperatures above 16 °C were not compromised, however, at 16 °C the cell density was decreased by 25% and growth rate decreased by 35%. The high rate of recombinant protein synthesis imposes a systemic stress on the cells which directly impacts their growth. In case of cells co-expressing recombinant chaperones GroEL/ES and aconitase, the biomass concentrations (cell growth) were found to be 10% higher than cells expressing only aconitase at all temperatures (Fig. 2B). The specific growth rate within 3 h after induction of the cells co-expressing GroEL/ES chaperone was slightly higher than the cells expressing only aconitase at temperatures of 30 °C and 25 °C indicating that the chaperones GroEL/ES were able to modulate this systemic stress at these temperatures (Fig. 2C). Estimation of aconitase expression levels in cells grown at various temperatures revealed that aconitase expression was highest at 37 °C which decreased with decrease in temperature. GroEL/ES co-expression had no major effect on aconitase expression, which remained nearly the same as in cells expressing aconitase in the absence of GroEL/ES co-expression (Table 1). At lower incubation temperatures, however, the GroEL/ES expression increased which can be explained by the slower rate of arabinose utilisation by the cells at lower temperatures.

**Table 1:** Effect of temperature on aconitase and GroEL/ES co-expression

Temperature (°C)	Aconitase expression (mg/g DCW) in cells expressing		GroEL expression (mg/g DCW) in *E. coli* cells	Fold increase in GroEL/ES expression as compared to 37 °C
	Aconitase	Aconitase and GroEL/ES		
37	24.03	23.78	14.67	–
30	23.68	22.90	24.40	1.66
25	20.93	20.13	59.49	4.06
16	18.42	18.07	84.19	5.74

**Fig. 3:**
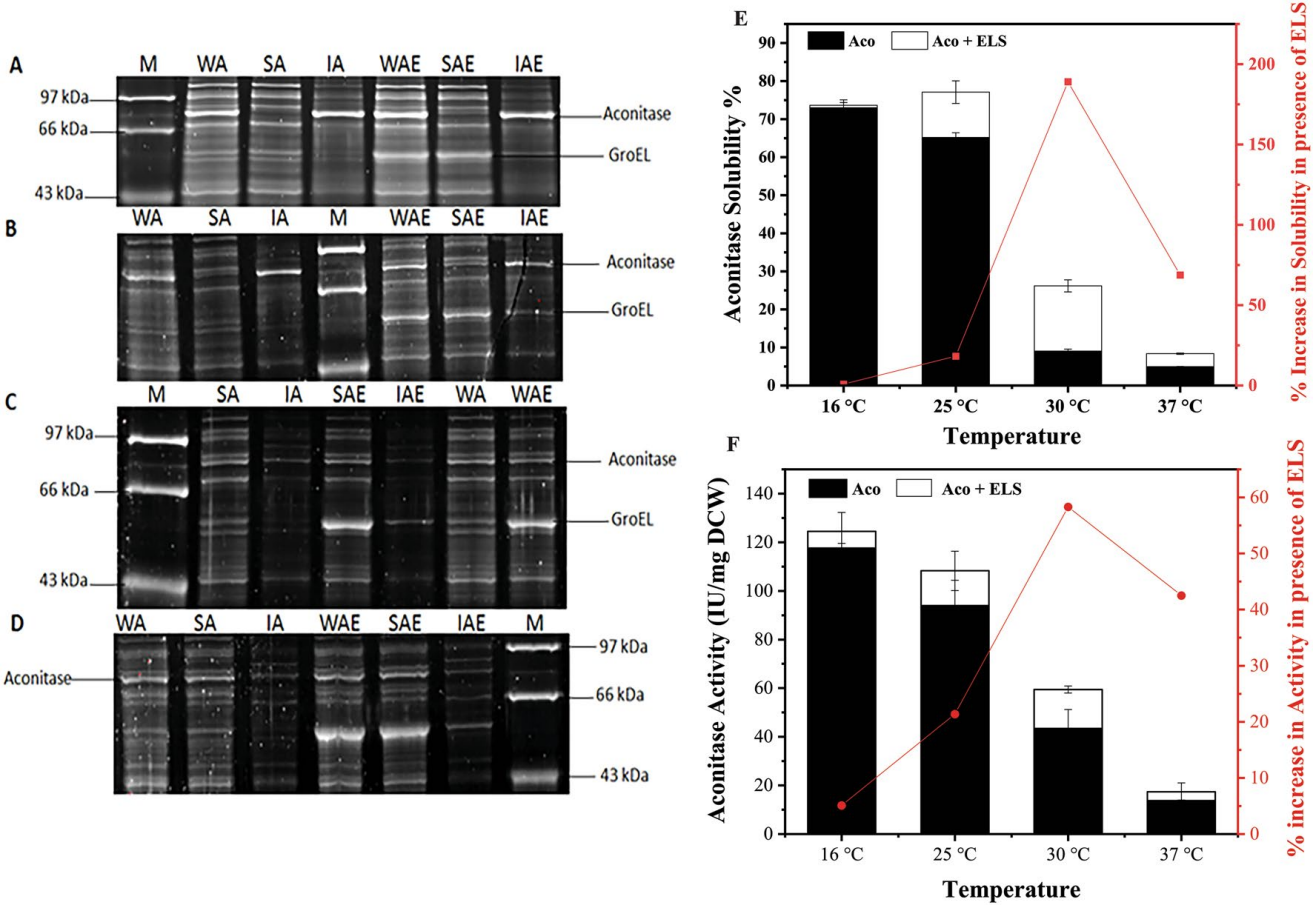
Consolidated SYPRO ruby stained 12% polyacrylamide gels loaded with cell lysates expressing recombinant aconitase in the absence and presence of co-expression of GroEL/ES grown at different temperatures. **A**–**D** Indicate the various cell fractions of induced BL21(DE3) cells grown at 37 °C, 30 °C, 25 °C and 16 °C respectively. The prefixes in the labels on the top of each lane in the gels, W, S and I denote the whole cell, soluble and insoluble fractions of the cell lysates. The suffixes in the labels A and AE denote the cells expressing aconitase in the absence and presence of concomitant expression of GroEL/ES. **E** The solubility of recombinant aconitase in BL21(DE3) cells grown at different temperatures during the absence and presence of co-expressed GroEL/ES chaperones and percent enhancement in aconitase solubility during GroEL/ES assisted folding. **F** The recombinant aconitase activities in BL21(DE3) cells at different temperatures during the absence and presence of co-expression of molecular chaperones and *percent enhancement in aconitase activity during GroEL/ES assisted folding*

The total expressed protein does not always reflect the amount of functionally active protein which is dependent on the ability of the protein to fold correctly. The functionally active protein can be estimated as the amount of protein which is in soluble form separated from the mis-folded non-functional protein aggregates, recovered as insoluble pellet fraction of the cell lysate. The SDS-PAGE gels images show the solubility analysis of the samples of the cell lysate as a whole and fractionated as soluble and insoluble components (Fig. 3A–D). The solubility of the recombinant aconitase was calculated as the fraction of the expressed protein present in the soluble cell lysate to the protein present in the total cell lysate. The aconitase yield increased rapidly reaching a peak within 2 h of induction at 37 °C. At 25 °C, the yield increased slowly and spread over a period of 10 h of induction proving the nascent polypeptide time to fold in the complex cellular environment. When aconitase expressing cells were grown at 16 °C, the solubility of the recombinant proteins was improved by 14-fold as compared to cells grown at 37 °C showing that the protein solubility tends to improve at lower temperatures. The solubility is further improved with GroEL/ES co-expression which helps in aconitase folding. The spontaneous folding of aconitase protein is severely hampered at 37 °C amounting to merely 5% which improves to just 9% in the presence of GroEL/ES co-expression. The most significant improvement in aconitase solubility was observed at 25 °C. The GroEL/ES assistance improved solubility at all temperatures except at 16 °C. Highest chaperone assisted folding was achieved from 9 to 27% at 30 °C (Fig. 3E).

Since only properly folded proteins are biologically functional, enzyme activity is the main determinant of a protein’s natural folding. Aconitase activity was improved by lowering the temperature but not to the same extent as solubility. At all temperatures the aconitase activity was enhanced in presence of GroEL/ES. The maximum change in folding and activity due to chaperone co-expression was recorded at 30 °C, with improvements in solubility and activity of 190% and 60%, respectively*.* Interestingly, the chaperone GroEL/ES expression level improved by 1.66-fold by lowering the temperature to 30 °C as compared to 37 °C without compromising the aconitase expression. The in vivo solubility of aconitase in the cells grown at 25 °C is found to be ~ 65% of the total aconitase expressed which is 11-fold higher compared to aconitase expressed in the cells grown at 37 °C. Similarly, the recombinant aconitase activity in the *E. coli* cells grown at 25 °C enhanced by sevenfold as compared to the activity in the cells grown at 37 °C. At 25 °C, the increment in the aconitase activity and solubility due to GroEL/ES co-expression is reported to be 21% and 18%, respectively (Fig. 3F).

### Microscopic imaging of morphologies of *E. coli* cells

**Fig. 4:**
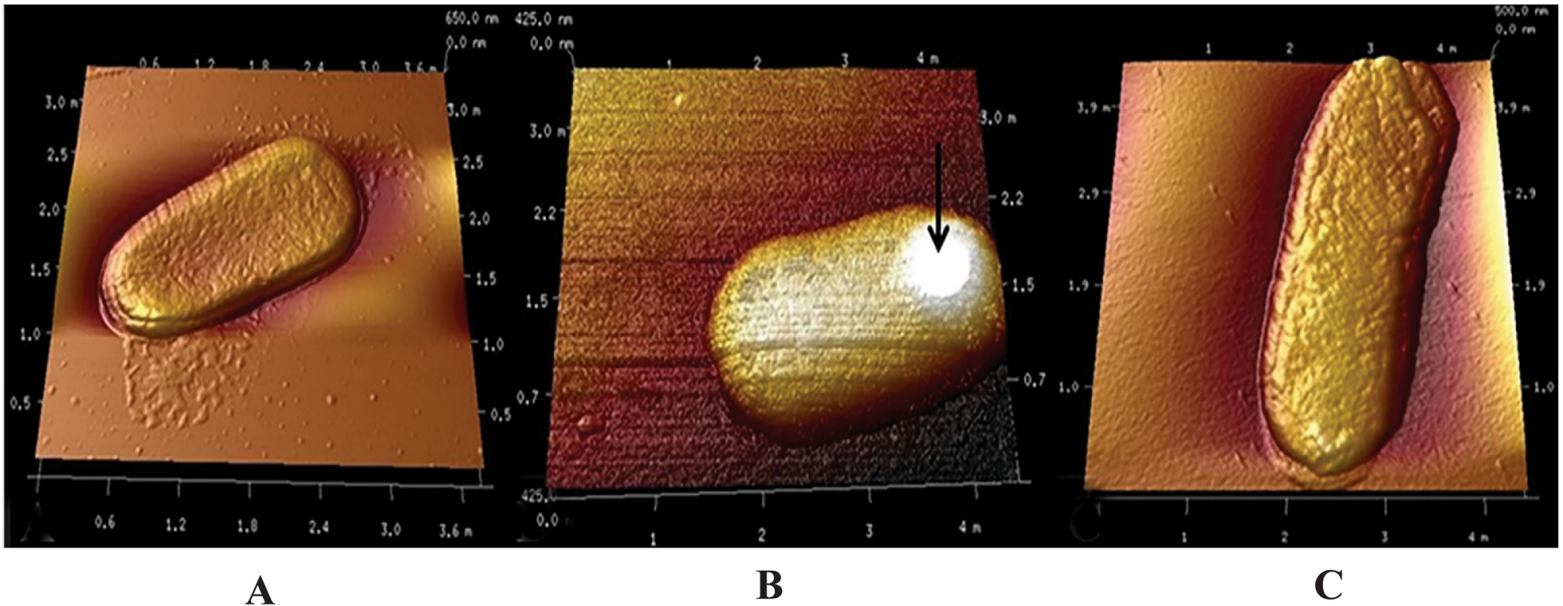
Atomic force microscopic images indicating the morphology of BL21(DE3) cells. **A** Depicts the morphology of uninduced BL21(DE3) cells grown at 37 °C. **B**, **C** Depict aconitase induced cells grown at 37 °C and 25 °C respectively. The black arrow indicates the inclusion bodies protrusion on the cell surface in the cells grown at 37 °C

The over-expressed recombinant protein tends to form inclusion bodies which reach to the micron size range. In order to study whether inclusion bodies have any effect on the cell morphology, the induced cells grown at 37 °C and 25 °C were studied by atomic force microscopy and compared against the control (uninduced) cells. The cells grown at 37 °C possessed areas of increased density denoting formation of inclusion bodies. They are mostly localized towards the poles which has been reported to be driven by the macromolecular crowding in the cytosol [14]. The presence of recombinant aconitase sequestered as inclusion bodies is clearly visible in *E. coli* cells grown at 37 °C both in the presence and absence of exogenous chaperones GroEL/ES. The black arrow in Fig. 4B indicate mass of inclusion body of expressed aconitase protein in the cells grown at 37 °C as a protrusion after 18 h of induction. No such significant formation of inclusion bodies was observed in the morphology of the cells expressing aconitase at lower temperature (Fig. 4C). Atomic force microscopy photos clearly highlight the contrast between cells grown at 37 °C and cells grown at 25 °C, where cells grown at 37 °C show a mass of inclusion bodies that is not seen in uninduced cells or cells grown at 25 °C.

### Effect of IPTG concentration on the aconitase expression and solubility

**Fig. 5:**
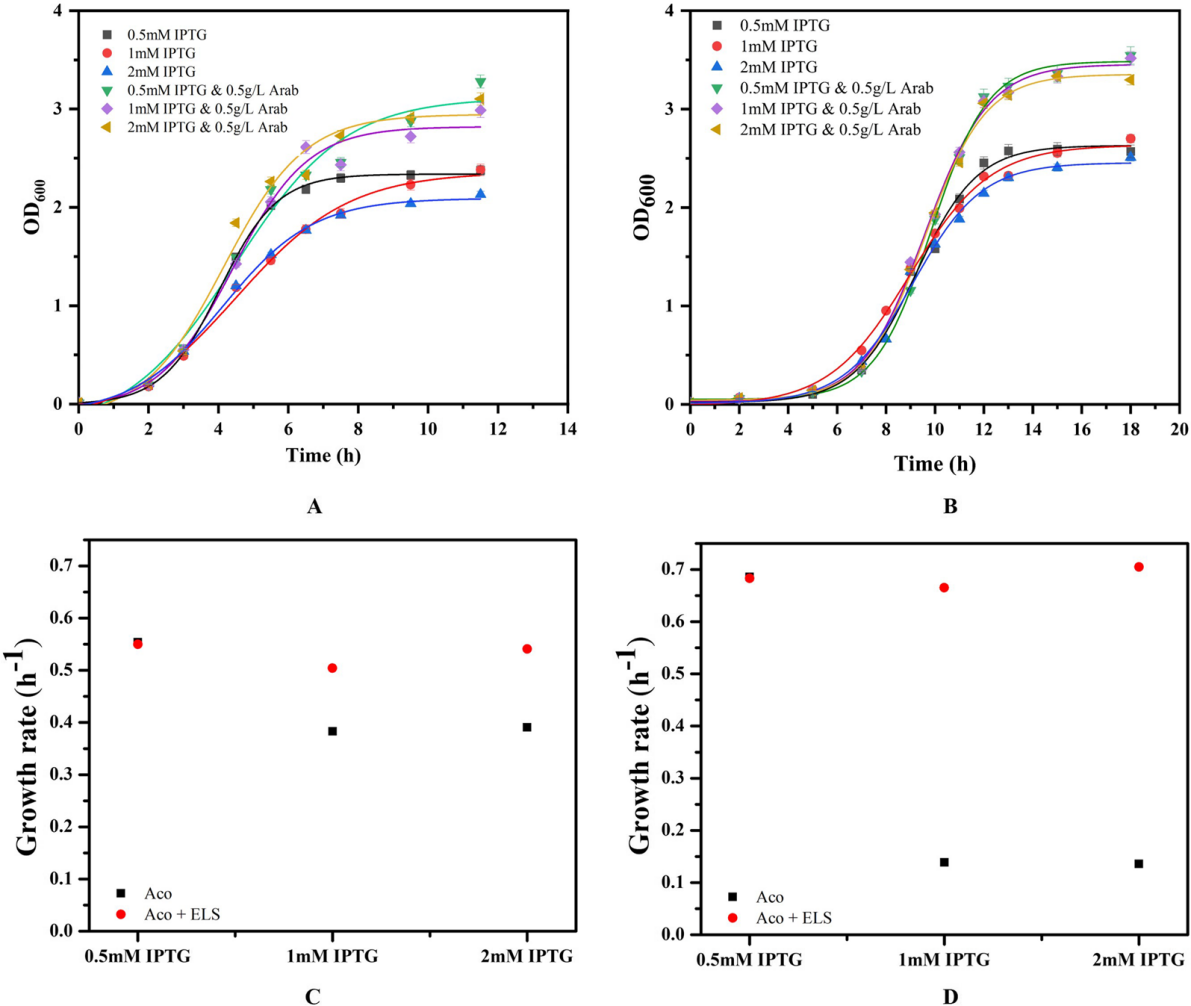
The effect of IPTG concentrations on biomass profiles of the induced cells. **A**, **B** Represent the *E. coli* cultures grown at 37 °C and 25 °C respectively, induced to express aconitase and GroEL/ES individually and in combination. The legend describes the three different IPTG concentrations used for the expression of the recombinant proteins in the *E. coli* cultures in the presence and absence of molecular chaperones co-expression. **C**, **D** Represent the specific growth rates of *E. coli* cultures grown at 37 °C and 25 °C respectively when induced with different IPTG concentrations

Aconitase expression induced from three different IPTG concentrations (0.5, 1 and 2 mM) were analysed in presence and absence of molecular chaperone co-expression at 37 °C and 25 °C. Growth rate tends to decrease with increase in IPTG concentration. The growth kinetics improves on GroEL/ES co-expression when the molecular chaperone rescues the expressed proteins from misfolding (Fig. 5A–D).

At 37 °C the exponential phase is short while at 25 °C the exponential phase is longer giving protein time to fold. This is also reflected in the solubility profile which improved at lower temperature. The aconitase expressed by cells grown at 25 °C showed better aconitase solubility as compared to those grown at 37 °C. It was found that the residual arabinose concentration got completely depleted within 3 h of induction with 0.5 g/L of arabinose when the cells were grown at 37 °C (data not shown). Therefore, enough GroEL/ES chaperones are not produced to fold the misfolded proteins leaving most of the protein in inclusion bodies at high temperatures. Under all conditions the aconitase solubility improved upon co-expression of GroEL/ES chaperone. While it improved very slightly at 37 °C, 15% enhancement in the aconitase solubility irrespective of the inducer concentrations was obtained at 25 °C.

**Fig. 6:**
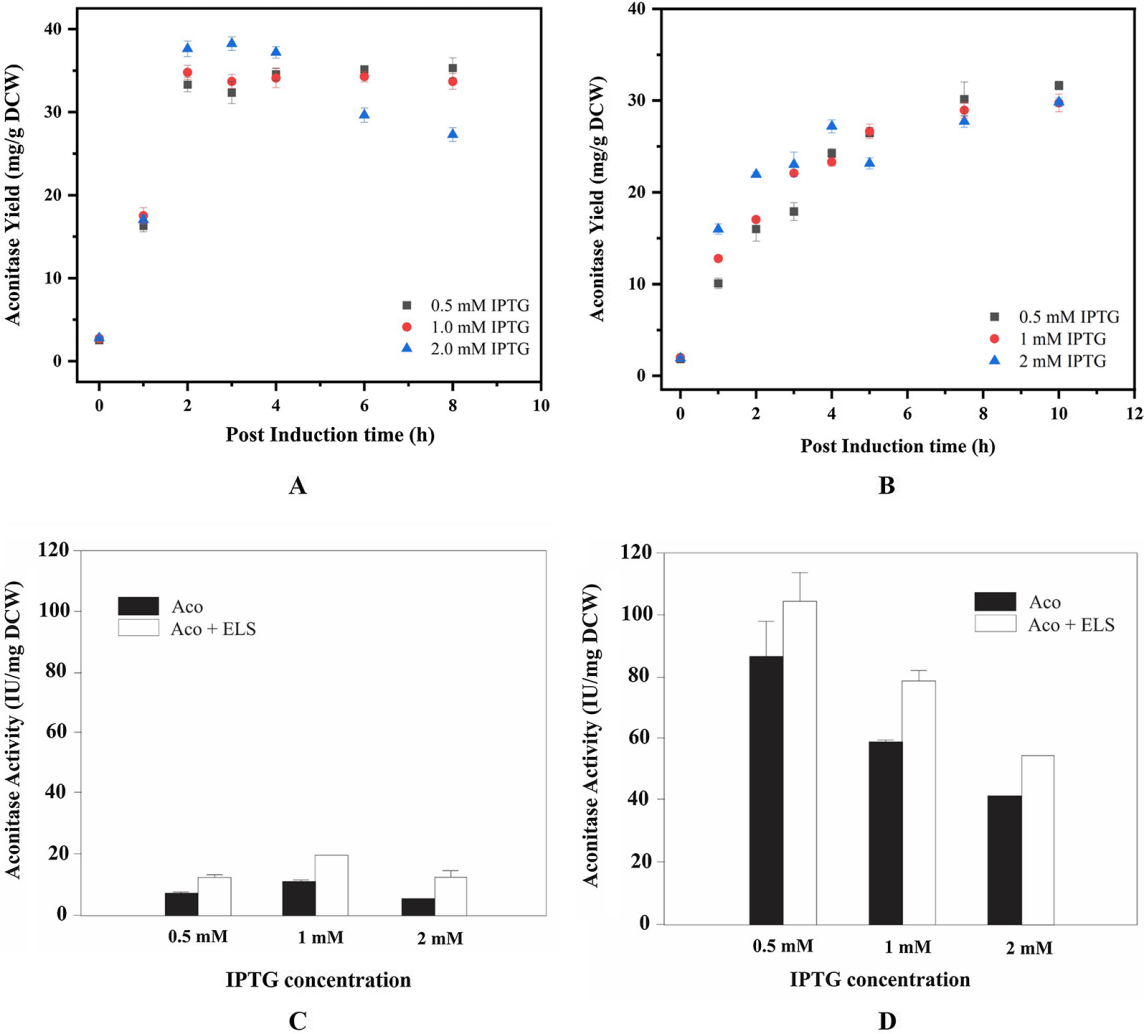
Aconitase yield profiles expressed in the recombinant BL21(DE3) cells induced with 3 different IPTG concentrations at different temperatures. **A**, **B** Denote the cells grown at 37 °C and 25 °C respectively. **C**, **D** Denote the effect of IPTG concentrations on the recombinant aconitase activity in cells grown at 37 °C and 25 °C during the absence and presence of co-expression of chaperones

Figure 6A and B depicts the aconitase yield obtained under different temperatures and varying inducer concentrations. A rapid increase in expression levels is observed in cells induced at 37 °C leading to large protein accumulation within 2 h of induction. Cells grown at 25 °C on the other hand exhibit a slower rate of production but the accumulation extended over a period of 10 h in the induction regime providing the nascent polypeptides enough time to fold in the complex cellular environment. It was seen that cells induced with high IPTG concentration (2 mM), showed a very high rate of synthesis during initial phase of induction at both the temperatures which retarded after 4 h.

It is well established that cells exhibiting high rates of protein synthesis incur high intermolecular interactions arising out of macromolecular crowding within the cells leading to formation of protein aggregates. Lower temperatures allow slow expression rates for longer durations. Also, the value of kinetic constants for intermolecular interactions are lower leading to less intermolecular interactions. The protein folding is further facilitated by molecular chaperones. As seen by the increment in aconitase activity by ~ 15% in cells grown at 25 °C at all inducer concentrations.

The solubility of the recombinant protein was not affected by inducer concentration irrespective of temperature or GroEL/ES expression. It was observed that only low temperature was the important factor and the protein exhibited improved solubility which was further improved in presence of GroEL/ES (Table 2). The aconitase activity reduced with increase in inducer concentrations. The cells treated with higher concentrations of IPTG at both temperatures showed detrimental effect on the aconitase activity. This demonstrates that when the expression rate increased, more of the produced protein lost functionality due to polypeptide misfolding. (Fig. 6C and D).

**Table 2:** Effect of IPTG induction on aconitase solubility in the recombinant BL21(DE3) cells during chaperone-mediated folding

IPTG concentration (mM)	Temperature (°C)	Aconitase solubility in *E. coli* cells expressing (%)	
		Aconitase	Aconitase and GroEL/ES
0.5	37	4.7	8.9
	25	64.1	76.4
1	37	5.1	8.5
	25	66.7	75.7
2	37	5.0	5.4
	25	65.7	74.9

### Titration of chaperone expression required for folding of aconitase

The co-expression of chaperones enables folding of aconitase but the expression of additional gene from an additional plasmid exerts further imbalance in the cellular metabolism and the proteins expressed compete for the tRNA pool of the cellular systems. This part of the experiments was carried out to demonstrate the effect of chaperone co-expression on the expression of the recombinant protein. The co-transformed *E. coli* cells were grown at 25 °C till OD_600_ of 0.6 to 0.8 and simultaneously induced with IPTG (0.5 mM) and varying arabinose concentrations ranging from 0.01 to 0.75 mg/mL for GroEL/ES expression to study its effect on aconitase expression, solubility and activity.

**Fig. 7:**
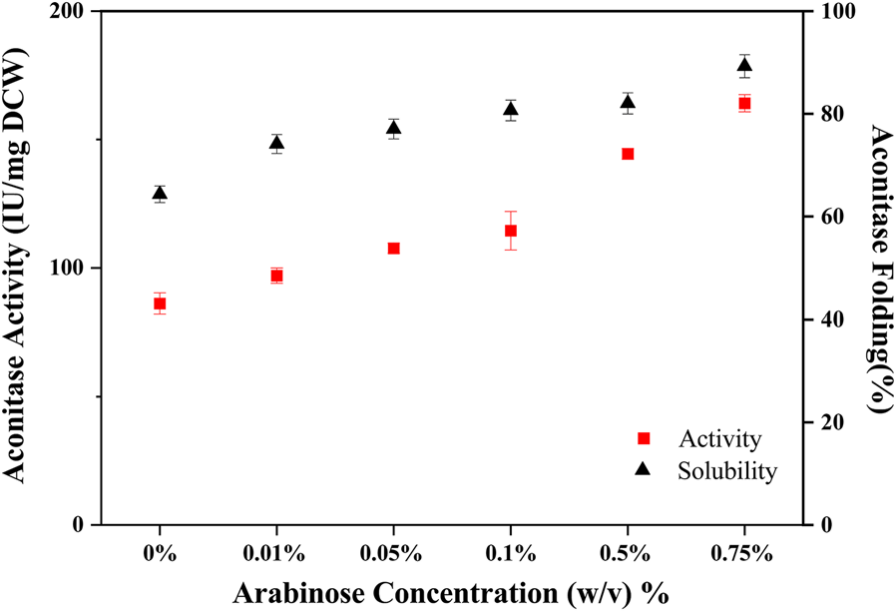
Effect of arabinose titration on aconitase activity and solubility in BL21(DE3) cells. The closed triangles and the closed squares represent the solubility and activity of the expressed aconitase, respectively when the cells transformed with the suitable plasmids were harvested after 14 h of induction

The GroEL/ES expression increased with increasing concentrations of arabinose reaching upto 75 mg/g DCW of GroEL/ES within 14 h of induction (Table 3). The aconitase expression was decreased by a maximum of 10% when both proteins were expressed at the same time. Compared to control cells with no exogenous GroEL/ES expression, the cells induced with increasing arabinose concentrations showed both increased aconitase activity and solubility. An 82% increase in aconitase activity reaching upto 145 IU/mg DCW was achieved when the recombinant proteins were expressed at 25 °C with 0.5% arabinose induction yielding ~ 60 mg/g DCW of exogenous GroEL/ES. The large improvement in aconitase activity at high GroEL/ES accumulation suggests that a lack of chaperones in the cellular environment prevents misfolded proteins from achieving their native structure (Fig. 7).

**Table 3:** Effect of arabinose titration on expression of aconitase and GroEL/ES in BL21(DE3) cells

Arabinose concentration % (w/v)	Aconitase yield (mg/g DCW)	GroEL/ES yield (mg/g DCW)
Control	10.73	–
0.01	8.8	17.1
0.05	9.11	25.6
0.1	8.9	28.4
0.5	9.33	58.3
0.75	8.98	74.10

### Effect of induction at different growth phases on the recombinant protein expression and activity

The time of induction during the fermentation plays a critical role in the final yield and the quality of the recombinant proteins in *E. coli*. The transformed *E. coli* cells were induced to express aconitase and GroEL/ES simultaneously at different cell densities. After 18 h of induction, the normalised quantity of cells was used to study the effect of induction at different growth rates. The induction of the culture at the early stage of growth, when the cells are growing rapidly, results in a low final biomass yield. However, if the culture was induced at later stages of growth, when the specific growth rate of the cells is lower, the specific product yield is reduced. It has been reported that the solubility of the recombinant product increased when the culture growing at lower temperature of incubation was induced to express at late-log growth phase [15].

When the culture was induced at low cell density, the induced cells grow at a fast growth rate and when induced at high cell density, the induced cells grow at a slow growth rate. The cells induced at slow growth rates show reduced recombinant aconitase expression and activity. The specific aconitase yield reduced by 40%, when the culture was induced at the optical density of 1.7. The reduction in the product yield at low specific growth rates may be due to the nutrient exhaustion and non-availability of precursor molecules for the polypeptide synthesis. Our results indicate that there is no appreciable change in the aconitase expression until the cell density of the culture goes beyond 1.3 (Table 4).

**Table 4:** Aconitase expression yield in the cells induced at various phases of growth

Cell density at the time of induction (OD_600nm_)	Aconitase expressed (mg/g DCW)
0.6	20.83
0.8	20.40
1.1	19.91
1.3	17.67
1.7	15.45

**Fig. 8:**
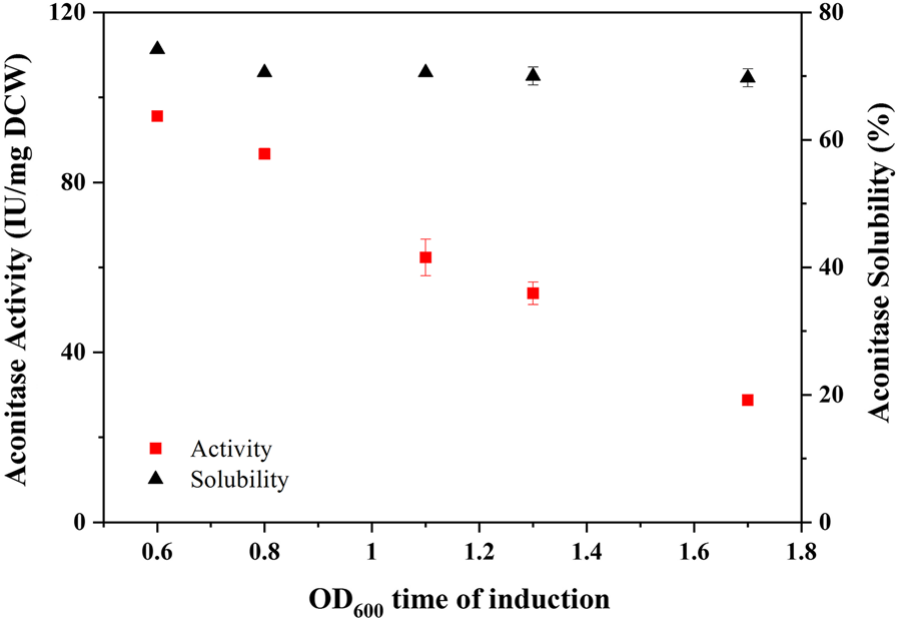
The effect of induction at different growth phases of the recombinant *E. coli* cells on aconitase activity and solubility during chaperone-assisted folding. The closed squares and triangles in the figure represent the activity and the solubility, respectively of the recombinant aconitase induced when the culture’s cell densities reached 0.6, 0.8, 1.1, 1.3 and 1.7 respectively

The aconitase activity reduction in the cells growing at slow growth rate indicates that the expressed proteins failed to attain native structural conformation. The recombinant aconitase solubility was not compromised when induced at higher cell density (Fig. 8). This indicates that the expressed proteins accumulate as soluble aggregates devoid of functionality. The optimal cell density for the induction of the recombinant aconitase and GroEL/ES to obtain soluble and functional recombinant proteins in BL21 (DE3) is reported to be 0.6.

### Effect of media components on recombinant aconitase activity and solubility during chaperone assisted folding

**Fig. 9:**
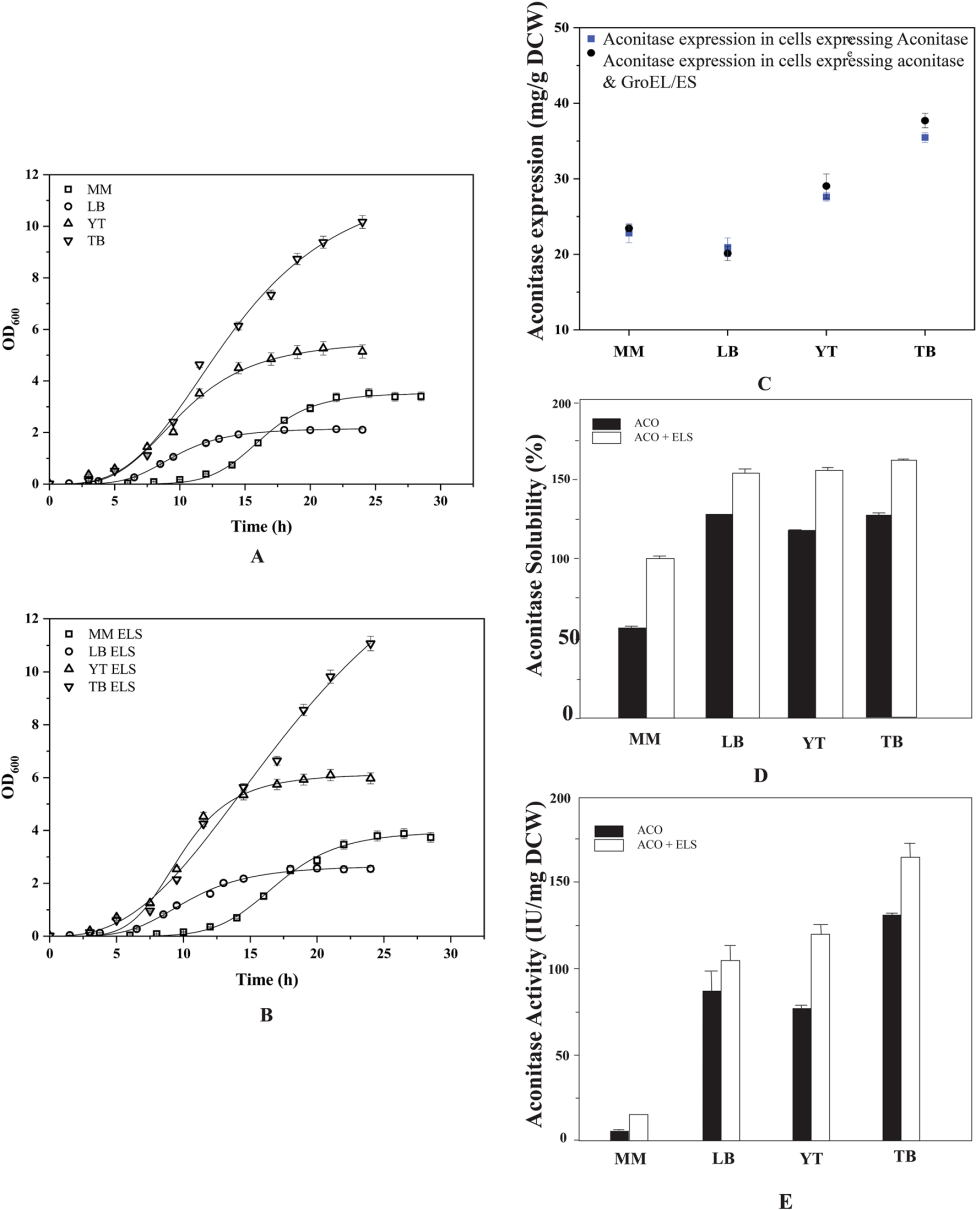
The effect of media components on the biomass profiles of the recombinant BL21(DE3) cells expressing recombinant proteins. **A** Denotes the cellular density profiles of the cells expressing only aconitase. **B** Denotes the cellular density profiles of the cells expressing aconitase and GroEL/ES simultaneously. The open squares, circles, triangles and inverted triangles indicate the recombinant cells grown at 25 °C in defined media, Luria broth, Yeast-Tryptone media and Terrific Broth respectively. **C** Depicts aconitase expression in mg/g DCW when grown in different types of media. **D**, **E** Depict the effect of media components on aconitase activity and aconitase solubility during the absence and presence of exogenous GroEL/ES expression

The transformed *E. coli* cells were grown at 25 °C in various media to see how media components affected the recombinant protein's expression, solubility, and functionality during GroEL/ES chaperone co-expression. The results in Fig. 9A and B show that the cells grown in media composed of richer nutrients displayed higher cell densities and growth rates compared to cells grown in chemically defined media. The aconitase expression was significantly higher in enriched media (LB, Yeast-Tryptone (YT), Terrific Broth (TB) as compared to defined media (MM) which showed comparatively less improvement on chaperone co-expression (Fig. 9C). The cells grown in media devoid of complex nutrient components displayed hindered aconitase solubility and activity. The aconitase solubility was very much affected in the cells grown in defined media where only about one-fourth of the expressed proteins were present in soluble form which was enhanced to 50% of the total expressed proteins upon chaperone co-expression (Fig. 9D). The aconitase activity is significantly lower as compared to cells grown in Luria Broth which could be due to the non-availability of the prosthetic group Fe_4_S_4_ for the aconitase activity in the cells grown in the defined media (Fig. 9E).

In cells grown in enriched medium, the specific aconitase activity was higher and about 60% of the expressed aconitase was in soluble form which increased to about 75–80% on chaperone co-expression. In minimal media, however, GroEL/ES assisted folding increased aconitase solubility by twofold. These set of experiments clearly demonstrate that media components are very important for the quality of the protein produced in the cells.

### Effect of osmolytes/compatible solutes augmented in the media

**Fig. 10:**
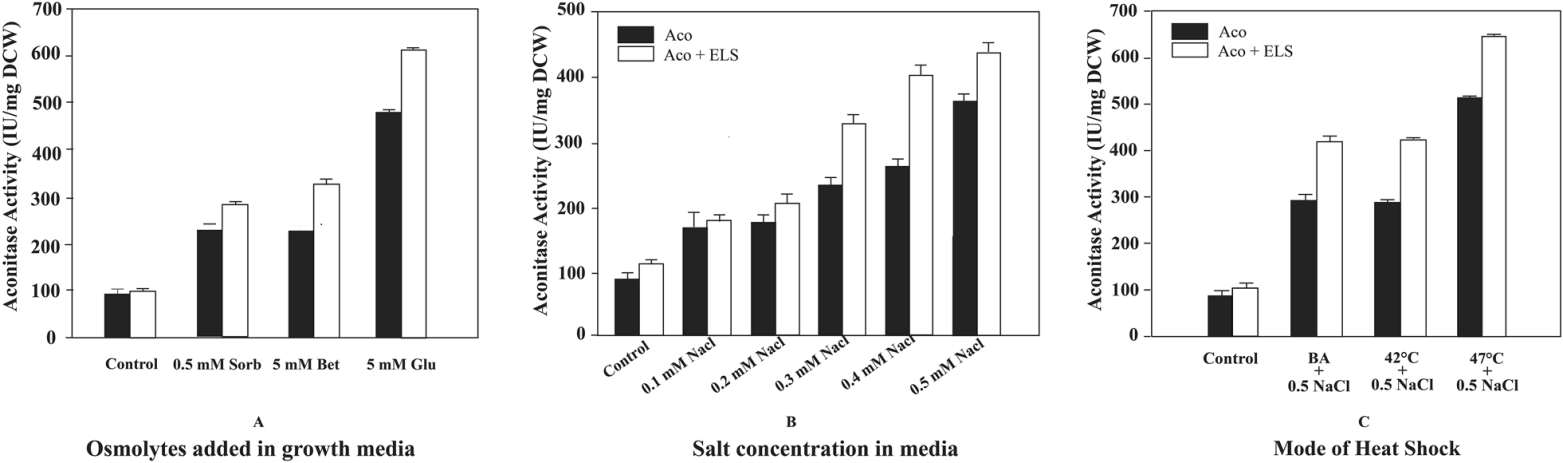
Effect of stress conditions on aconitase activity. **A** Effect of osmolytes/compatible solutes augmented in the complex media. The control cells were not exposed to osmolytes and were grown in LB media. **B** Effect of osmotic stress on recombinant protein activity during chaperone assisted folding. **C** The effect of pre-induction heat shock stress on the recombinant aconitase activity expressed in BL21(DE3) cells grown at 25 °C. BA stands for benzyl alcohol treated cells (chemically induced heat stress condition)

Osmolytes are known to have a direct impact on the solubility of proteins by assisting in protein folding and preventing protein aggregation. When recombinant *E. coli* cells were grown in media supplemented with osmolytes (sorbitol, betaine and glutamate), an improved enzymatic activity was observed with all the three osmolytes as compared to controls without the osmolytes (Fig. 10A). These experiments demonstrated that the over-expressed protein was able to acquire bio-functionality due to attainment of native structure confirmation in presence of these osmolytes. The presence of glutamate increased aconitase expression by 1.6-fold while also resulting in a fivefold increase in activity as compared to control, which can be attributed in part to its metabolizable nature.

### Effect of osmotic stress in media on recombinant *E. coli*

In reference with the existing research, the *E. coli* cells, when subjected to osmotic stress, are triggered to synthesize osmolytes like betaine and trehalose through their cellular metabolism, resulting in their accumulation inside the cell in millimolar concentrations [16]. Therefore, in order to induce osmolyte synthesis through osmotic stress, the cells were subjected to increased salinity in the growth media. The recombinant cells previously adapted to grow in osmotic stress exhibited higher growth rates compared to cells inoculated from un-adapted cultures. The cells grown under high osmotic stress conditions showed a decrease of ~ 30% in growth rates and ~ 60% in cell density (Table 5). The osmotic stress conditions induced by increased salinity decreased the aconitase expression while increasing the recombinant aconitase activity. The *E. coli* cells adapted to grow at higher salinity (0.5 M of NaCl) reportedly accumulate high concentrations of betaine and trehalose [17] resulting in improved aconitase activity as compared to control cells grown without any osmotic stress.

**Table 5:** Effect of osmotic stress in the growth rate, cell density and aconitase yields

Salinity in media	Growth rat^#^ (h^−1^)	Harvest OD_600_	Aconitase (mg/g DCW)
Control	0.55	2.14 ± 0.05	20.93
0.1 M NaCl	0.53	1.60 ± 0.02	20.75
0.2 M NaCl	0.48	1.34 ± 0.04	20.17
0.3 M NaCl	0.45	0.94 ± 0.09	19.53
0.4 M NaCl	0.43	0.81 ± 0.02	18.74
0.5 M NaCl	0.40	0.67 ± 0.01	17.57

^#^The growth rate of the cells in the initial growth phase of the culture

The increment in the aconitase activity was further improved by ~ 5 folds with the co-expression of GroEL/ES at higher osmotic stress conditions (Fig. 10B). While the aconitase activity was improved, there was no significant change in the solubility of recombinant proteins due to osmotic stress in *E. coli* cells grown at 25 °C. This indicates that the increment in the activity of recombinant aconitase is due to the enhancement in the folding of the proteins accumulated as soluble aggregates devoid of functionality due to the presence of the endogenous osmolytes and GroEL/ES.

### Effect of pre-induction heat shock on the recombinant aconitase activity in presence of chaperone-assisted folding

The *E. coli* cells, when subjected to heat shock are induced to produce some constitutive heat shock proteins in order to overcome the heat shock stress. These proteins include some foldases which assist in the folding of mis-folded proteins and proteases which degrade the aggregated moieties. In order to see the effect of pre-induction heat shock on the solubility of aconitase protein, the transformed cells were subjected to a thermal shock by incubating the cultures at elevated temperatures (42 °C and 47 °C) for 20 min before induction. In another set of experiments the cells were subjected to chemically induced heat shock stress by addition of benzyl alcohol in the LB media. This triggers a heat shock like response by fluidizing the cell membranes [18]. The heat shock induced by benzyl alcohol (BA) and thermal shock at 42 °C resulted in about twofold increase in the aconitase activity and thermal shock at 47 °C resulted in about threefold increase in aconitase activity as compared to the control cells grown in LB at 25 °C. When the cells were subjected to a combination of osmotic stress along with heat stress (chemically induced or thermal shock at 42 °C), the aconitase activity increased by twofold as compared to cells experiencing only heat shock. The activity was further improved to threefold in presence of GroEL/ES. The highest aconitase activity with fourfold and fivefold increase in absence and presence of GroEL/ES was obtained with cells subjected to pre-induction heat shock at 47 °C indicating that the host cell’s heat shock protein machinery was participating in the folding of recombinant aconitase (Fig. 10C). In terms of solubility, cells exposed to benzyl alcohol-induced heat shock had lower solubility than cells exposed to physical heat shock stress at 42 °C, while aconitase solubility was not significantly altered when cells were exposed to both physical and chemical heat treatment at 47 °C. A profound sevenfold increase in recombinant aconitase activity was observed in cells subjected to a pre-induction heat shock at 47 °C and osmotic stress in combination with GroEL/ES chaperone assisted folding.

## Discussion

Proteins are a class of molecular machines that are engaged in structural and transport components, have enzymatic properties, and play a role in cell signalling. As the proteins are in distinct shapes, performing varied functions, each protein owns a unique 3D structure innate with its distinct amino acid sequence. The amino acid sequence dictates its primary structure, yet in order to perform its activities the proteins need to fold precisely into an accurate three-dimensional form. The process of protein folding is remarkably efficient and conserved through the evolutionary process [19, 20]. Upon translation the proteins are released into the complex cellular environment among several other proteins and biomolecules where several factors come together to catalyse the folding process which is unique to each protein. While advances in recombinant DNA technology have enabled the cloning and expression of almost any protein, yet the fully functional state can only be achieved when the proteins are provided with uniquely optimized conditions for its folding. Understanding the folding process is the most fundamental step towards production of these proteins under non-native conditions such as an industrial set-up. Due to the long amino acid sequence the number of possible conformations is very large, however the native structure involves interaction between residues that are stable and lowest energy structures. Chaperones are molecular catalysts that help the protein to reach this conformation [21]. Cellular stress conditions bring about episodes of massive protein misfolding. Under such conditions the level of molecular chaperones substantially rises providing evidence that they rescue misfolded proteins providing them with another chance to refold. This type of intervention requires energy, which is why many of the chaperones require ATP to function [22]. Over-expression of heterologous proteins also imposes stress like conditions in *E. coli* which is one of the most extensively utilised expression host organisms for the creation of recombinant proteins. This results in a considerable amount of the produced protein accumulating as an insoluble mass devoid of biological function. The cellular chaperones are insufficient to fold such large amounts of proteins. Therefore, the co-expression of molecular chaperones along with the recombinant proteins is an effective strategy for enhancing the solubility of proteins [23–25]. *E. coli* also possess an in-built mechanism to cope with physiological stress conditions. When bacterial cells are subject to stress such as osmotic and heat-shock they adapt themselves to overcome it by synthesizing bio-molecules and endogenous heat-shock proteins that can evade the harsh effects and help in normal functioning of the cells [26]. The influence of various process parameters on the expression, solubility, and activity of recombinant proteins during co-expressed chaperone-mediated folding was investigated in this study. GroEL is one of the best characterized molecular chaperones, possessing a cavity which holds the unfolded protein away from the cellular environment, folds it and then releases it [27]. Yeast mitochondrial aconitase, an 82 kDa monomeric Fe_4_S_4_ cluster containing enzyme has been used as a model protein in this study as it was found to fold both in vivo and in vitro through multiple rounds of binding and release with both GroEL and GroES. The recombinant protein aconitase is severely aggregation prone under the physiological growing conditions of *E. coli* and are rescued from forming massive non-functional, non-native aggregates by assistance of GroEL/ES chaperones [13].

In this work the gene for aconitase was cloned in pET29 under the strong T7 promoter and expressed in BL21(DE3). The transformed cells were retransformed with pGro7 for expression of GroEL/ES. The optimal growth temperature plays the most critical role on the growth rates, cellular biomass, recombinant protein expression, solubility and activity thus defining the quantity and quality of the exogenous protein produced in the cellular milieu. The rate of synthesis of the foreign proteins is rapid at higher temperature which leads to the quick accumulation of the nascent polypeptides without reaching its native structural conformation [28]. The large mass of intermediate proteins with exposed hydrophobic patches invites for various non-covalent interactions both weak and strong. Morphologically aggregates of the synthesized proteins as inclusion bodies were clearly visible in the *E. coli* cells grown at 37 °C both in presence and absence of chaperones while no such aggregates were observed at 25 °C. Efficiency of the strong promoters is usually affected at low temperatures decreasing the rates of expression. The low enthalpy and the reduced molecular movements give the protein moieties the time-period for its folding to attain the native structural conformation before the cytosolic environment become crowded and saturated with misfolded polypeptides [29]. The slow rates of cellular metabolism at low temperatures directly contributes to the reduction in the transcriptional and translational rates. In *E. coli* cells grown at lower temperatures, exhibited slower growth rates and reduced cellular densities but the aconitase folding increased as shown in the improved solubility and enzymatic activity. The increase in the enzymatic activity was more pronounced in the cells grown at 25 °C, when the aconitase solubility was 65% of the total expressed protein. Furthermore, the *E. coli* cells grew comfortably at 25 °C even though a decline in the substrate utilisation rate has been reported. The chaperoning activity of GroEL/ES in the cells grown at 30 °C showed better increment in the recombinant protein activity and solubility than at any other incubation temperatures. The enhanced ability of the GroEL/ES to hydrolyse the ATP molecules at 30 °C, is the reason for its improved chaperoning activity. In order to utilize the benefit of chaperone co-expression optimally, the cells need to be incubated at 30 °C. Increasing the amount of GroEL/ES by exogenous expression, improved the solubility and activity of aconitase indicating the demand for the co-expressed chaperones in the cytoplasm for assisting the proper folding of the nascent polypeptides. It also indicates that the endogenous chaperones present at the physiological conditions are not sufficient for the folding of the recombinant proteins. Further, combining induction at early-log phase cultures with low post-induction temperatures for protein induction resulted in even more active soluble proteins [15].

When genes are expressed from strong promoters like T7 promoters, the rate of mRNA synthesis is not rate limiting, rather, it is cellular translational reactions that are rate limiting. The cellular translational efficiency is determined by the availability of ribosomes, the tRNA abundance in the cells and also on the physiological state of the cells. The competition for tRNA pool for the native and the recombinant protein synthesis and the degradation of rRNA molecules leading to lesser number of functional ribosomes result in the gradual decline in rate of recombinant protein synthesis and a progressive decline in the growth rate of cells [30, 31]. Lowering the rate of transcription and translation by reducing the quantity of inducer employed also resulted in a drop in polypeptide production rates to give protein moieties ample time to fold [32]. A higher concentration of IPTG had a negative effect on aconitase activity, indicating that more protein lost functionality by misfolding at high expression levels. A 2 mM IPTG caused toxic effects in the cells and inhibits the polypeptides from attaining the native structural conformation directly reflecting in the loss of recombinant enzyme activity.

When the cellular resources are already being strained by internal protein synthesis and protein synthesis from a plasmid, introducing a plasmid for chaperone expression adds to the burden, and as a result aconitase expression drops. A titration of arabinose concentration was carried out to obtain a better balance where the extra plasmid improved aconitase solubility without lowering its expression too much. At 25 °C, an optimal arabinose concentration for regulated expression of GroEL/ES at 0.5% improved aconitase activity by 82%. The presence of plasmids in addition to cell’s internal protein synthesis requires abundant nutrients to make up for the burden. When LB medium was used, the cells grew slowly, which might be due to a shortage of glucose in the media, which would allow for quicker development. The defined media components are not sufficient enough for the recombinant cells to produce the heterologous products in the functional form. Enriched media containing complex nutrient components such as yeast extract and tryptone can offer an environment for the cellular machinery to alleviate stress caused by nutrient deficiency, allowing it to manufacture large amounts of functional proteins. The solubility and activity also improved in enriched media as compared to defined media. Under all conditions expression of molecular chaperones improved protein folding. In the present experiments, use of enriched media (TB) was found to be better based on the yield of functional protein production. But, since we wanted to learn about the process of cellular folding of the aggregation-prone protein, aconitase, in one of the most widely used media, we chose LB. It has various advantages, such as being readily available and being less expensive. In the future, we hope to do similar tests in medium that produce a high yield of recombinant protein. In addition, future research should look into the various factors that can improve recombinant protein production and incorporate them into the minimal media, which would allow for better control over media composition in order to optimise a process for large-scale functional recombinant protein production.

The analysis of *E. coli* stress response is not just limited to gene expression, system level stress adjustment may also take place. Compared to the general transcript level response, the metabolic response is more specific especially during the early stress adaptation phase. One of the key responses is energy conservation by decreasing central carbon metabolism which reflects in the slowed growth rates. Downregulation of genes involved in translation and ribosome synthesis is a common response [33]. Specific responses include those targeted at repair functions, such as cellular production of osmolytes against an osmotic stress and biogenesis of chaperones as part of the heat shock response when native proteins are misfolded after being exposed to higher temperatures. When osmolytes/ chemical chaperones like betaine and sorbitol were added to *E. coli* cultures during the production of recombinant proteins, protein solubility improved to a large extent. These osmolytes are small molecules like betaine and trehalose, that maintain a positive turgor and reduce the water loss in the cells [34]. Osmolyte synthesis was induced by giving osmotic shock to cells through exposure to high salt concentrations also resulted in similar response. This assumption was made based on a previous publication [16], and while none of the investigations to determine osmolyte production were carried out in the current study, the recombinant protein's solubility and function were observed to have improved as a result of the osmotic stress. The mechanism of protein folding in the presence of osmolytes involves interaction between the exposed highly hydrophobic backbones of the proteins with the osmolyte which destabilizes the non-native structure of the protein [35]. Apart from this, the ionic environment in the cytoplasm prevents the intermolecular interactions between proteins inhibiting soluble aggregate formation. In a similar fashion transcription factors, chaperones, proteases, and other heat-shock proteins provide a survival advantage to the heat stressed organism. When the cells are grown at physiological temperatures, the sigma factor, σ^32^ is bound to the heat shock chaperone proteins like DnaK/J/GrpE and GroEL/ES and is present in inactive form [36]. However, when the cells are exposed to heat shock, the bound σ^32^ is released from the chaperone-sigma factor complex and can activate transcription of the genes under its control. The heat shock response is generated by the rapid accumulation of misfolded recombinant proteins in the cytoplasm. The free σ^32^ factor triggers the synthesis of many heat shock proteins like DnaK/J/GrpE and GroEL/ES [37]. The upregulation of these chaperones promotes the folding of the cytoplasmic intermediate-folding species. The other proteins that are upregulated are the proteases ClpP and Lon and small heat shock proteins IbpA and IbpB [38, 39]. The proteases degrade the recombinant proteins accumulated as misfolded species and inclusion bodies [40]. The small heat shock proteins bind to the insoluble aggregates and protect them from degradation [41, 42]. In an attempt to trigger conditions similar to the natural heat stress response the cells were exposed to a pre-induction heat shock and a chemically induced heat shock. An improvement in aconitase activity was found to increase with increasing heat shock temperature. This suggests that the host cell’s internal machinery was participating to refold the proteins to their functionally active forms. This response along with inclusion of osmolytes and co-expression of GroEL/ES led to upto fivefold increase in activity. The strategies mentioned above can be very effective in combination to achieve higher yields of functional recombinant proteins.

## Conclusions

The growth conditions of the host cells expressing the recombinant aconitase plays an important role in determining the quality of the product produced by the cells. Growth conditions that include growth temperature and media components were analyzed for their effect on protein functionality. At 25 °C, recombinant aconitase solubility and activity increased by 11-fold and sevenfold respectively compared to cells grown at 37 °C due to slow down in the transcription rates that allowed proteins time to fold correctly. The media components played a very important role in the quality of protein. Recombinant cells grown in enriched media demonstrated higher aconitase activity and solubility as compared to cells grown in defined media. Both aconitase (IPTG) and GroEL/ES (arabinose) inducer concentrations were optimized for improved functional protein to achieve a balance between transcription rates that do not negatively affect protein folding.

The cells subjected to physiological stress like osmotic and heat shock stresses showed fivefold and threefold increase in the recombinant aconitase activity respectively compared to the cells grown in no stress environment. The cells subjected to the combination of osmotic and heat shock stress exhibited sixfold increase in the aconitase activity. The recombinant aconitase solubility in the cells subjected to the osmotic and heat stresses was found to be 70% of the total expressed aconitase. This implies that the improved activity is due to the proper folding of the soluble aggregates to attain the native structure configuration under the presence of endogenous chaperones. The endogenous chaperones expressed and the osmo-protectants synthesized during the physiological stimuli facilitate the folding process by providing the non-native structures a plethora of options for folding assistance. Under all experimental conditions, the co-expression of chaperone GroEL/ES and a low post-induction temperature improved both solubility and activity of the aconitase protein. The physiological properties like the specific growth rate of the cells were also improved in the cells co-expressing GroEL/ES suggesting that chaperones helped to alleviate the cellular stress imposed by heterologous protein expression.

## Methods

### Plasmids, strains and growth conditions

The functional gene encoding the yeast mitochondrial aconitase *aco1* (Accession No. CP046092.1) was amplified from the parent plasmid pQE60Aco (gifted by Dr. Sabine Rospert, Germany). The plasmid pET29a (Novagen, Madison, Wis.) was used for gene expression. The plasmid pGro7 expressing GroEL and GroES was purchased from Takara Inc., Japan. All primers were designed using the GeneRunner®software. The pGro7 plasmids carry the chaperone genes (GroEL/ES) downstream of the araB promoter. This allows the plasmids to be used with *E. coli* expression systems that utilize ColE1-type plasmids containing the ampicillin resistance gene as a marker. A complete list of plasmids and primers used in this study is enlisted in the *additional file* (Additional file 1: Table S1and S2). The commercial *E. coli* strain DH5α was used for general cloning and plasmid maintenance and *E. coli* BL21(DE3) [(F^−^*ompThsdS*_*B*_*(r*_*B*_^*−*^*m*_*B*_^*−*^*) recA1 gal dcm*(DE3)] (Invitrogen, USA) was used as host for recombinant yeast mitochondrial aconitase (rAco) expression in this study. The transformed *E. coli* cells were routinely maintained in Luria Broth (LB) containing 50 µg/mL of kanamycin and 20 µg/mL of chloramphenicol.

### Cloning of aconitase gene in pET29a vector

The mature 2.4 kb aconitase gene (*aco1*) was PCR amplified from the parent vector pQE60Aco in order to obtain higher expression and to ensure plasmid compatibility with the GroEL/ES expressing vector. The PCR was performed with an initial denaturation of 5 min followed by 35 cycles of 92 °C for 45 s, 56 °C for 1 min, and 72 °C for 2 min, and a final elongation step for 10 min. The amplified gene was re-cloned in the pET29a vector between NdeI and XhoI restriction sites using standard procedures and the resulting plasmid construct after ligation was designated as pETAco. The vector was transformed into DH5α and the transformed clones were confirmed by restriction digestion and DNA sequencing.

### Development of *E. coli* co-expression system

The *E. coli* strain BL21(DE3) [(F^−^*ompThsdS*_*B*_*(r*_*B*_^*−*^*m*_*B*_^*−*^*) recA1 gal dcm*(DE3)] (Invitrogen, USA) was used as host for gene expression. Transformation with pETAco was carried out followed by re-transformation with the plasmid pGro7 to express both aconitase and GroEL/ES simultaneously as per standard protocol. The co-transformed BL21(DE3) cells were induced with 0.5 mM of sterile IPTG and 0.5 g/L of l-arabinose to induce aconitase and GroEL/ES expression. Protein expression was verified on the 12% SDS-PAGE gel.

### Growth profiles and specific growth rate of cells

Samples collected at various stages of cell growth at regular time intervals were centrifuged at 5000 rpm for 5 min and the pellets were washed and dispersed in 0.85% (w/v) saline solution. The optical density of the suitably diluted samples was recorded at 600 nm in DU800, Beckman Spectrophotometer where 1 A.U. spectrophotometrically corresponded to 0.3715 g DCW/L of *E. coli* cells. The specific rate of growth (µ_max_) was determined from the slope of the growth curve plotted between biomass OD_600nm_ and fermentation time on the semi-log graph.

### Cell fractionation and protein quantification

The cells grown under different process conditions were harvested after 12 to 18 h of induction. Normalized quantities of cells were centrifuged at 10,000 rpm at 4 °C for 10 min. The cell pellet was resuspended in10 mL of cold cell lysis buffer (50 mM Tris, 50 mM KCl, 15 mM Tricarballylic acid, 0.1 mM Fe^2+^, 10 mM PMSF and 10 mM DTT) and incubated once for 30 min. The constant cell disruptor system of TS series (Constant System Ltd., UK) pre-cooled to 4 °C was used for cell lysis at a constant hydraulic pressure of 20psi. The cells suspension was passed twice through the cell disruptor to ensure complete cell lysis. The cell lysate was centrifuged at 12,000 rpm for 30 min at 4 °C. The aconitase expression levels were determined from the whole cell samples collected either during the sampling points or at the end of the harvest. For determining the protein solubility, the cells pellet was lysed to release the intracellular contents in the lysis buffer. The soluble components were separated by centrifugation of the cell lysate. The supernatant and the pellet were resuspended in the SDS loading buffer and analysed on a 12% SDS-PAGE. Equal volume of the samples was loaded on the SDS-PAGE gel along with 3 different concentrations of BSA as internal standards in each gel. The gel was subjected to staining with SYPRO Ruby fluorescent gel stain (Sigma, USA) and washed with the washing gel solution. The stained gel image was captured under UV lamp illumination using Bio-Rad Molecular Imager® Gel Doc XR + Imaging Unit. The absolute quantification of band intensity was estimated using Image Lab® software in Bio-Rad Molecular Imager Gel Doc XR + unit by densitometric analysis using known amounts of BSA as internal standards.

$${\text{Solubility (\%) = }}\frac{{\text{Aconitase Band Intensity in soluble fraction of cell lysate}}}{{\text{Aconitase Band Intensity in fraction of cell lysate}}} \times 100$$

### Aconitase enzyme assay

Aconitase activity of the soluble fraction of cell lysate was carried out at 20 °C in 1 mL reaction mixture containing 20 mM of DL-tri sodium isocitrate (buffered with 90 mM Tris, pH 7.4) as the substrate for the enzyme. The formation of *cis*-aconitic acid was measured at 240 nm spectrophotometrically after the addition of 50 µL of the cell lysate in the reaction mixture and recorded every 10 s for 10 min using the kinetics/time application in DU 800 Beckman Coulter spectrophotometer. The slope (ΔC/Δt) which represents the rate of formation of the product (*cis*-aconitate) was determined from the initial linear region of the curve. An extinction coefficient (ε) of 3.6 mM^−1^ cm^−1^ was used for *cis*-aconitic acid at 240 nm. One unit (IU) activity of aconitase corresponds to 1µmole of *cis*-aconitate produced from DL-tri sodium isocitrate per minute [43, 44]. Aconitase activity (IU/ mg DCW) was determined from the following equation:

$${\text{Aconitase activity }} = \frac{{\left( {\Delta {\text{C}}/\Delta {\text{t}}} \right) \, \times {\text{ V}}_{{\text{r}}} \times {\text{ D}}}}{{{\upvarepsilon } \times {\text{V}}_{{\text{s}}} \times {\text{d}}}}$$

where (ΔC/Δt) is the slope of the activity assay curve in mM/min; ε is the extinction coefficient of *cis*-aconitic acid in mM^−1^ cm^−1^; V_r_ is the reaction mixture volume in mL (1 mL); V_s_ is the volume of the cell lysate used in mL (50 µL); D is the correlation between OD_600nm_ and cell dry weight in per g DCW; d is the path length in cm.

### Effect of temperature on the cell density, expression, solubility and activity of aconitase in recombinant *E. coli* cells

The transformed cells were grown in 100 mL LB media at different temperatures (37 °C, 30 °C, 25 °C and 16 °C) respectively at an agitation of 200 rpm. The growth of the cells was monitored and optical density was recorded at regular time intervals. At mid-log phase (between OD_600_ of 0.6–0.9) the cells were induced with 0.5 mM IPTG and 0.5 g/L of l-arabinose to express aconitase and GroEL/ES respectively. The cells were harvested and fractionated into soluble and insoluble pellet fraction. 12% polyacrylamide gels loaded with cell lysates expressing recombinant aconitase in the absence and presence of co-expression of GroEL/ES grown at different temperatures was stained using SYPRO ruby dye. The intensity of the aconitase band on the whole cell, soluble and insoluble fractions of the cell lysates was determined using BSA as internal standards.

### Microscopic imaging of morphology of *E. coli* cells

The induced samples of *E. coli* cells expressing aconitase and GroEL/ES grown at different temperatures (i.e., 37 °C and 25 °C) were centrifuged and pellets were resuspended in 0.5 M Tris (pH 7.5) buffer. 5 µL of the sample was laid on freshly peeled mica sheet (1 cm × 1 cm) and allowed to air dry. The samples were subjected to tapping mode analysis in atomic force microscope (Biocatalyst, Bruker Inc., USA).

### Effect of inducer concentration on cell growth and expression

The *E. coli* BL21(DE3) cells transformed with pETAco and pGro7 were grown in 100 mL LB media supplemented with appropriate antibiotics at two different temperatures of 37 °C and 25 °C. In one set of experiments, the cells were induced with 0.5 mM, 1 mM and 2 mM of IPTG concentrations to express only aconitase. To study the effect of chaperone assisted folding of the recombinant aconitase, in another set, the cells were also induced with 0.5 g/L of l-arabinose to express exogenous GroEL/ES. The expression profiles of aconitase genes were analysed on a 12% SDS-PAGE gel.

### Arabinose titration

The co-transformed *E. coli* cells were inoculated from a primary culture into 6 shake flasks with 50 mL LB medium supplemented with appropriate amounts of antibiotics (50 µg/mL of kanamycin and 20 µg/mL of chloramphenicol). When OD_600nm_ reached 0.6 to 0.8, the cells were induced with 0.5 mM IPTG and arabinose concentrations of 0% to 0.75% (w/v) for GroEL/ES induction. The cultures were harvested after 14 h of induction. The specific yields of aconitase and GroEL/ES were determined from the band intensities on the SDS-PAGE gels against BSA bands intensities of known concentrations.

### Effect of induction at different growth phases

The transformed BL21(DE3) cells were grown in 6 × 100 mL of media in 500 mL flasks at 25 °C at 220 rpm. The cultures were induced at different cell densities (0.6, 0.8, 1.1, 1.3 and 1.7) with 0.5 mM IPTG and 0.5 g/L of l-arabinose. The cultures were harvested after 18 h of induction. A normalised quantity of the culture was centrifuged at 4 °C and the cell pellet was stored at − 20 °C until further analysis. The cells were lysed after re-suspension of the cell pellet in cold lysis buffer and aconitase activity and solubility were determined.

### Effect of growth media components on the recombinant *E. coli*

To study the effect of media components on the gene expression, the cells were grown at 25 °C at 200 rpm in the following media: (a) LB (10 g/L casein hydrolysate, 5 g/L yeast extract,5 g/L NaCl), (b) Yeast-Tryptone (YT) (10 g/L yeast extract, 16 g/L tryptone and 5 g/L sodium chloride), (c) Terrific Broth (TB) (24 g/L yeast extract, 12 g/L tryptone, 0.4% glycerol to which 2.31 g/L KH_2_PO_4_ and 12.54 g/L K_2_HPO_4_ were added after autoclaving separately) and (d) chemically defined media (MM) (0.5 g/L NaCl, 1 g/L NH_4_Cl, 3 g/L K_2_HPO_4_, 6 g/L of KH_2_PO_4_, 2 mL of 1 M MgSO_4_ per L, 5 g/L glucose and trace elements (10 mg/L CaCl_2_.2H_2_O; 0.5 mg/L ZnSO_4_·7H_2_O; 0.25 mg/L CuCl_2_·2H_2_O; 2.5 mg/L MnSO_4_·H_2_O; 1.75 mg/L CoCl_2_·6H_2_O; 0.125 mg/L H_3_BO_3_; 2.5 mg/L AlCl_3_·6H_2_O; 0.5 mg/L Na_2_MoO_4_·2H_2_O; 5 mg/L Thiamine; 40 mg/L FeSO_4_·7H_2_O). The primary culture was prepared in 50 mL in the corresponding media with the cells grown in 5 mL of LB with the antibiotics kanamycin and ampicillin. 1 mL of the primary culture was used to inoculate a secondary culture of 100 mL sterile media. Induction for protein expression was done with 0.5 mM of IPTG and 0.5 mg/mL of arabinose in MM and LB media at OD_600nm_ of 0.6–0.8 and in TB and YT media at OD_600nm_ of 1–1.5.

### Effect of presence of co-solutes/osmolytes in the growth media on the recombinant aconitase activity and solubility

The transformed *E. coli* cells were grown in LB media supplemented with the osmolyte sorbitol. When the OD_600nm_ reached 0.6 to 0.8 the cells were induced with IPTG and arabinose. In another set of experiments the cells grown in LB media amended with 0.5 M sodium chloride (LBS) were treated with 5 mM of potassium glutamate (Glu) and 5 mM of betaine (Bet) when the culture reached OD_600nm_ of ~ 0.5. The cultures were induced for expression of aconitase and GroEL/ES by adding 0.5 mM IPTG and 0.5 mg/mL of arabinose respectively when OD_600nm_ reached 0.6 to 0.8.

### Effect of osmotic stress on the recombinant aconitase activity and solubility

To study the effect of osmotic stress on the activity and solubility of recombinant aconitase, the transformed BL21(DE3) cells were grown in 5 mL of LB media supplemented with 0.5 M sodium chloride to adapt the cells to osmotic stress. 1 mL of this overnight grown culture was used to inoculate 100 mL LB media supplemented with salt in a concentration range of 0.1 M to 0.5 M and incubated at 25 °C and 200 rpm. The growth profile was determined and cells were induced at mid-log phase with IPTG and arabinose to express the recombinant proteins aconitase and GroEL/ES.

### Effect of pre-induction heat shock stress response on the recombinant *E. coli*

The cells were grown in 100 mL of LB and LBS media. On reaching an OD_600nm_ of 0.6–0.8, the flasks were transferred to water bath maintained at 42 °C/ 47 °C in two separate sets of experiments. After incubating for 20 min, the culture was brought back to 25 °C until the time of harvest. For a chemically induced heat shock response, the culture was treated with 10 mM benzyl alcohol and incubated for 20 min before induction with IPTG.

## Supplementary Information

**Additional file 1: Fig S1.** Cloning of ACO1 gene in pET29a vector. Lane M: 1 kb DNA marker ladder; Lane 1: PCR amplification of gene encoding yeast mitochondrial aconitase, Lane 2 The recombinant plasmid pETAco digested with NdeI and XhoI enzymes showing the gene inserted as “pop-out”. **Table S1.** Plasmids used in this study*.* **Table S2.**. Primer sequence designed for PCR amplification of aconitase gene.

## Data Availability

The datasets used and/or analysed during the current study are available from the corresponding author on reasonable request. All data generated or analysed during this study are included in this published article [and its Additional file 1].

## Abbreviations

*E. coli*: *Escherichia coli*
IPTG: Isopropyl β-d-1-thiogalactopyranoside
DCW: Dry Cell Weight
LB: Luria Broth
YT: Yeast-Tryptone broth
TB: Terrific Broth
MM: Minimal media (chemically defined media)
OD: Optical density
