# Supplementary material for: Multimodal approaches for the improvement of the cellular folding of a recombinant iron regulatory protein in E. coli
Source: Microb Cell Fact. 2022 Feb 5;21:20. doi: 10.1186/s12934-022-01749-w (PMC8818239; doi:10.1186/s12934-022-01749-w)
Supplement: Supplementary file 1 — Additional file 1: Fig S1. Cloning of ACO1 gene in pET29a vector. Lane M: 1 kb DNA marker ladder; Lane 1: PCR amplification of gene encoding yeast mitochondrial aconitase, Lane 2 The recombinant plasmid pETAco digested with NdeI and XhoI enzymes showing the gene inserted as “pop-out”. Table S1. Plasmids used in this study. Table S2.. Primer sequence designed for PCR amplification of aconitase gene. [file 12934_2022_1749_MOESM1_ESM.docx]

**Additional file**

**
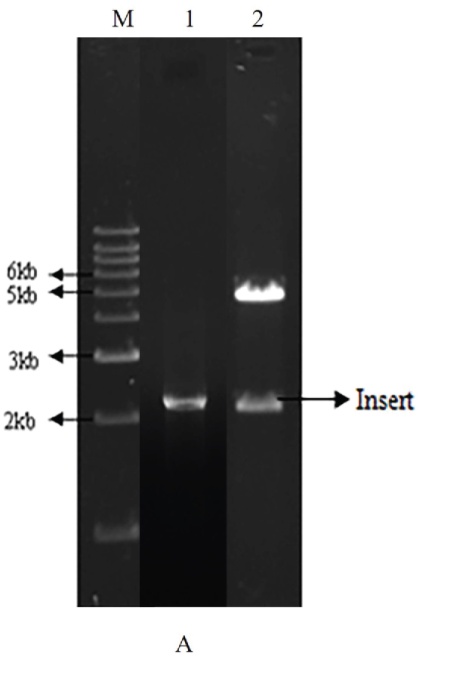
**

**Fig S1:** Cloning of ACO1 gene in pET29a vector. Lane M: 1kb DNA marker ladder; Lane 1: PCR amplification of gene encoding yeast mitochondrial aconitase, Lane 2 The recombinant plasmid pETAco digested with NdeI and XhoI enzymes showing the gene inserted as “pop-out”.

**Table S1. Plasmids used in this study**

| Plasmid | Gene cloned | Promoter | Antibiotic resistance | References |
| --- | --- | --- | --- | --- |
| pQE60Aco | Aconitase | *Lac* | Ampicillin | Chaudhuri, *et al.*, 2001 |
| pETAco | Aconitase | T7*lac* | Kanamycin | This study |
| pGro7 | GroEL/ES | *arab* | Chloramphenicol | Nishihara, *et al.*, 1998. |

**Table S2 Primer sequence designed for PCR amplification of aconitase gene**

| Primers | Sequence ^#^ |
| --- | --- |
| Forward Primer | 5’ TTA**CATATG**CTGGATTCAAAAGTCAACC 3’ |
| Reverse Primer | 5’ TTT**AAGCTT**TTATTTCTTCTCATCGGC 3’ |

^#^ The bold sequences are the restriction digestion recognition sites for *Nde*I and *Xho*I in the forward and reverse primers respectively
